# Thought Suppression in Primary Psychotic Disorders and Substance/Medication Induced Psychotic Disorder

**DOI:** 10.3390/ijerph18010116

**Published:** 2020-12-26

**Authors:** Cosmin O. Popa, Razvan Predatu, Wesley C. Lee, Petronela Blaga, Eliza Sirbu, Adrian V. Rus, Alexander Clark, Cristiana Cojocaru, Alina Schenk, Vitalie Vacaras, Simona Szasz, Simona Muresan, Cristina Bredicean

**Affiliations:** 1 Department of Ethics and Social Sciences, George Emil Palade University of Medicine, Pharmacy, Sciences and Technology, 540142 Tirgu-Mures, Romania; cosmin.popa@umfst.ro; 2 International Institute for the Advanced Studies of Psychotherapy and Applied Mental Health, Babeș-Bolyai University, 400015 Cluj-Napoca, Romania; petronelablaga@psychology.ro; 3 Department of Clinical Psychology and Psychotherapy, Babeş-Bolyai University, 400015 Cluj-Napoca, Romania; 4 Department of Social and Behavioral Sciences, Southwestern Christian University, Bethany, OK 73008, USA; wesley.lee@swcu.edu (W.C.L.); adrian.rus@swcu.edu (A.V.R.); 5 Doctoral School “Evidence-based Assessment and Psychological Interventions”, Babeș-Bolyai University, 400015 Cluj-Napoca, Romania; 6 Doctoral School of George Emil Palade University of Medicine, Pharmacy, Sciences and Technology, 540142 Tirgu-Mures, Romania; eliza_sirbu@yahoo.com (E.S.); cristiana-manuela.cojocaru@umfst.ro (C.C.); schenkalina@yahoo.com (A.S.); 7 College of Education and Professional Studies, University of Central Oklahoma, Edmond, OK 73034, USA; clarkalex616@gmail.com; 8 Neurology Department, Cluj Emergency County Hospital, 400006 Cluj-Napoca, Romania; vvacaras@umfcluj.ro; 9 Department of Rheumatology, Physical and Rehabilitation Medicine, George Emil Palade University of Medicine, Pharmacy, Sciences and Technology, 540142 Tirgu-Mures, Romania; szasz_fc@yahoo.com; 10 Department of Internal Medicine, George Emil Palade University of Medicine, Pharmacy, Sciences and Technology, 540142 Tirgu-Mures, Romania; 11 Department of Neuroscience, Victor Babeș University of Medicine and Pharmacy, 300041 Timișoara, Romania; brediceancristina@gmail.com

**Keywords:** thought suppression, first-episode psychosis, primary psychotic disorders, substance/medication induced psychotic disorder, automatic thoughts, stress-related growth, cognitive behavioral therapy

## Abstract

Introduction: First episode-psychosis (FEP) represents a stressful/traumatic event for patients. To our knowledge, no study to date has investigated thought suppression involved in FEP in a Romanian population. Our objective was to investigate thought suppression occurring during FEP within primary psychotic disorders (PPD) and substance/medication induced psychotic disorders (SMIPD). Further, we examined the relationship between thought suppression and negative automatic thoughts within PPD and SMIPD. Methods: The study included 30 participants (17 females) with PPD and 25 participants (10 females) with SMIPD. Psychological scales were administered to assess psychotic symptoms and negative automatic thoughts, along a psychiatric clinical interview and a biochemical drug test. Results: Participants in the PPD group reported higher thought suppression compared to SMIPD group. For the PPD group, results showed a positive correlation between thought suppression and automatic thoughts. For the SMIPD group, results also showed a positive correlation between thought suppression and automatic thoughts. Conclusions: Patients with PPD rely more on thought suppression, as opposed to SMIPD patients. Thought suppression may be viewed as an unhealthy reaction to FEP, which is associated with the experience of negative automatic thoughts and might be especially problematic in patients with PPD. Cognitive behavioral therapy is recommended to decrease thought suppression and improve patients’ functioning.

## 1. Introduction

According to the DSM-5, psychotic disorders are debilitating psychiatric disorders, in which the patient loses touch with reality, has delusional ideation, auditory and sometimes even visual hallucinations, disorganized thinking/speech, extremely abnormal behavior, as well as negative symptoms [1]. As a psychiatric diagnosis, psychosis is divided into several distinct categories such as schizophrenia, schizotypal (personality) disorder, brief psychotic episode, substance/medication induced psychotic disorder, and psychotic disorder due to another medical condition. In the scientific literature, there is a clear distinction between primary psychotic disorders (PPD) (i.e., schizophrenia, brief psychotic episode, schizotypal disorder, and delusional disorders) and substance/medication induced psychotic disorder (SMIPD), as well as psychotic disorder due to another medical condition [2,3]. The main difference between PPD and SMIPD is that psychotic symptoms in SMIPD are caused by substances or drugs, while in PPD they are generally triggered by negative life events and psychological trauma [4,5]. In addition, positive symptoms and cognitive dysfunctions (e.g., memory, visual attention, and language) remit faster in patients with SMIPD than in patients with PPD [6,7]. Demographic, familial, and clinical differences can also be observed between studies of patients admitted to a psychiatric inpatient service with PPD and those with SMIPD. For example, patients with PPD show more severe psychiatric symptoms including suicidal ideation, violent behavior, and lifetime suicide attempts [8]. On the other hand, patients with SMIPD are predominantly male who are involved in a marital or conjugal relationship, in contrast to PPD [9]. Furthermore, patients with SMIPD are less likely to have a family history of psychosis, have higher levels of insight, show more severe hostility or anxiety than those with PPD, and they are more likely to have a forensic history, a diagnosis of antisocial personality disorder, or trauma histories [2]. In addition to their history, SMPID patients most likely had an unsupportive family coupled with parents who struggled with alcohol and drug addiction. Hence, patients with PDD and SMIPD present a separate clinical picture with different precipitating factors, and different symptoms may be related to a different experience of control, varying levels of experienced distress, and different coping mechanisms employed (cognitive and behavioral) by patients [8,9].

Psychotic symptoms are disturbing personal experiences (e.g., delusional symptoms and auditory hallucinations); therefore, patients are prone to use thought suppression as a cognitive coping strategy towards delusional thoughts, as well as towards the associated negative automatic thoughts and emotions related to these unpleasant internal experiences [10,11,12]. Thought suppression—the deliberate attempt not to think of something—is a strategy of cognitive control, consciously initiated, often in the service of emotion regulation when thoughts create unpleasant emotions [13]. As a cognitive mechanism, thought suppression appears in conditions in which patients try to control the content of intrusive/negative thoughts, rejecting or suppressing them [13,14]. Therefore, if a person struggles with unwanted thoughts and tries to remove these thoughts (e.g., “My future is bleak”), the unwanted thoughts will resurface with amplified intensity. Moreover, other unwanted thoughts may be associated with the initial content (e.g., “I’m worthless”) [15]. In other words, thought suppression has been found to have a rebound effect. Specifically, individuals who engage in thought suppression are prone to the replication of that cognitive content over time [16]. From this perspective, thought suppression represents a maintenance and exacerbation factor in many psychological disorders (e.g., depression and anxiety), increasing the frequency of unwanted/intrusive thoughts [17]. Despite its efficacy in the short term, longer periods of thought suppression are associated with higher distress as a result of prolonged efforts to maintain mental focus [18].

A similar cognitive pattern might emerge for patients with psychotic symptoms. Aversive events, such as delusions, hallucinations, and other associated aversive internal experiences (e.g., negative thoughts and emotions) may trigger suppression in these individuals, which may further worsen their mental disorder [19]. Consequently, during treatment, patients are encouraged to reassess or reduce the distress related to delusional content and negative automatic thoughts. Alternatively, patients try to ignore these thoughts or suppress them [20]. Patients with psychosis may use thought suppression as a cognitive coping strategy (e.g., to reduce the distress associated with the delusions and/or auditory hallucinations); however, as stated earlier, with a potential rebound effect [21]. There is evidence that psychotic patients also use cognitive reappraisal frequently to reduce negative emotions, such as anxiety or depression [22]. However, research indicates that there is a preference for suppression over cognitive reappraisal among psychotic patients who experience positive symptoms [23]. Psychotic symptoms can be a natural target for thought suppression in schizophrenia, since this coping strategy tends to be applied to private experiences with high social disapproval or whose content is related to harming self or others [24]. Thus, thought suppression is most widely viewed as a maladaptive strategy used by individuals with psychosis to cope with their psychotic symptoms, as well as with other associated aversive internal experiences, such as negative automatic thoughts or negative emotions [25].

Indeed, besides the association between thought suppression and psychotic symptoms, another important feature of thought suppression is its relationship with negative automatic thoughts, which are strongly associated with depression [15] or/and anxiety disorders [16]. Automatic thoughts represent rigid thought patterns (e.g., “My future is bleak” or “I’m worthless”), which are not developed because of cognitive deliberation or debate [26], resulting in a high level of emotional discomfort [27]. It is common for individuals with psychosis to have an increased level of negative automatic thoughts, which are often associated with anxiety and depression, that are frequently co-occurring with psychosis [24,28]. Therefore, automatic thoughts are relevant to the exacerbation and complication of psychosis, such as developing an associated depression or anxiety disorder. However, we are not aware of studies capturing the relationships between thought suppression and negative automatic thoughts in psychosis, especially in a Romanian population. This will further point towards the complexity of this condition and the need to address multiple cognitive mechanisms (such as negative automatic thoughts and thought suppression) potentially underlying its development, maintenance and exacerbation.

### The Overview of the Present Study

In the last decades, thought suppression was specifically described in conjunction with obsessive-compulsive disorder [29], major depressive disorder [30], as well as other maladaptive strategies such as self-injurious thoughts and behaviors [31]. However, other researchers also focused on this strategy of controlling thoughts by suppression in psychosis. Specifically, previous research has addressed this strategy from the perspective of coping/metacognitive mechanisms, with results indicating that thought suppression occurred in psychotic disorders, such as schizophrenia [32,33,34,35,36,37,38]. Indeed, the cognitive control of thoughts, intrusive thoughts control, or thought suppression in psychotic disorders have been investigated in studies in various countries, such as US, Germany, and UK [17,32,39]. All these studies pointed towards the fact that thought controlling or avoidance strategies, such as thought suppression, were associated with the experience of psychotic symptoms and other emotional difficulties (e.g., depression and anxiety) in individuals with psychosis. However, despite preliminary evidence on these relations in various countries, to our knowledge, no study to date investigated thought suppression in first episode-psychosis (FEP) in a Romanian population. In addition, to our knowledge, this is the first study focusing on two categories of psychosis: PPD and SMIPD in a Romanian population. Given this background, the first objective of the present study was to investigate the cognitive strategy of thought suppression in FEP patients with two distinct diagnoses, specifically PPD and SMIPD. In this regard, we explored if there are significant differences regarding the use of thought suppression between these groups. The second objective was to investigate the relationship between thought suppression and negative automatic thoughts within the two groups. Given that thought suppression was previously conceptualized as a maladaptive strategy, we expected that an increased level of thought suppression will be associated with higher levels of negative automatic thoughts in both groups.

## 2. Materials and Methods

### 2.1. Participants and Procedure

The study was conducted from May 2019 to August 2020 at the Mures County Hospital, Psychiatric Clinic No. 1 from Tirgu Mures, Romania. Written consent was obtained from participants after they were informed about the study and its implications. Consent was also obtained from appropriate Romanian authorities. Data protection was ensured. The study was approved by the Institutional Ethics Committee of the Mures County Hospital from Tirgu Mures under the number 7843/28.05.2019.

The study included two groups of patients diagnosed with a psychotic disorder. The first group (target group) consisted of 30 participants (17 females, 13 males) diagnosed with a PPD (Mage = 27.3; SD = 6.92). The second group (control group) consisted of 25 (10 females, 15 males) participants diagnosed with SMIPD (Mage = 23.24; SD = 4.16).

The inclusion criteria were (1) patients had to be diagnosed with PPD and to experience a FEP; (2) patients had to be diagnosed with SMIPD and to experience a FEP; (3) age between 20–50 years; (4) patients from the PPD group had to test negative on a biochemical drug test; (5) patients from the SMIPD group had to test positive on a biochemical drug test; and (6) the total score of the Positive and Negative Syndrome Scale (PANSS) had to be 30 or over for each patient. The exclusion criteria were: (1) patients who presented a second psychotic episode; (2) psychosis caused by medical conditions; (3) patients with schizophrenia; and (4) delusional disorder. In our study, our focus was to control confounds as much as possible and include patients with FEP. Thus, patients with schizophrenia and delusional disorder were excluded due to their chronicity. Such patients may present a different pattern of vulnerability than those with FEP (due to the chronicity), thus were excluded from the present study.

The working procedure included identifying patients with psychosis to establish the study groups. In order to establish a diagnosis of psychotic disorder, the psychiatric clinical interviewing was based on the DSM-5 diagnostic criteria and the PANSS, which were conducted within the emergency room and the outpatient clinic of the psychiatric hospital. The biochemical testing related to the consumption of psychoactive substances was also performed. Biochemical testing followed immediately after the psychiatric clinical interview and the application of the PANSS (before hospitalization), including all patients with a diagnosis of a psychotic disorder. This is the standard protocol in psychiatric clinics, based on the recommendations of the National Institute for Health and Care and Excellence (NICE) Guide for the psychotic disorders, regarding the clinical assessment and physical health of patients who present with FPE [40]. A specific diagnostic of PPD and SMIPD was established based on three important clinical aspects: (1) the conclusions of the psychiatric clinical interviewing, in concordance with the DSM-5 criteria; (2) the PANSS scores; and (3) the final results of the biochemical tests. Immunochromatographic assays identified the presence of drugs/psychoactive substances in urine. After the psychiatrist explained the purpose of the urinalysis, individuals signed an informed consent document. The administration and interpretation of the test was rapid (between 4 and 10 min) and the result was interpreted binary, negative or positive. Patients with FPE who scored 30 or over at PANSS, who met the diagnostic criteria for PPD, and tested negative on the biochemical test were assigned to the PPD sample. Patients with FPE who scored 30 or over at PANSS, who met the diagnostic criteria for SMIPD, and tested positive on the biochemical test were assigned to the SMIPD sample. Patients in both study groups (N = 55) were selected from a total of 123 patients. There were 59 participants not included in the study because they did not meet the inclusion criteria. Furthermore, 9 participants in the PPD group dropped out of the study, not willing to further continue it. At about 7–12 days after the first day of hospitalization, patients completed the following psychological questionnaires: The White Bear Suppression Inventory (WBSI) and The Automatic Thoughts Questionnaire (ATQ). Clinical interviewing was conducted only by psychiatrists, while the PANSS and the psychological questionnaires were administrated by both psychiatrists and psychologists from the psychiatric clinic. The basic condition stated was that patients must be able to understand Romanian language and be able to answer items on the written psychological questionnaires. All the patients included in the study were treated using antipsychotic medication which was prescribed by a psychiatrist.

### 2.2. Measures

#### 2.2.1. The Positive and Negative Syndrome Scale (PANSS)

The PANSS [41] is a 30-item scale which was developed to obtain a measure of positive and negative symptoms in schizophrenia, as well as measuring general psychopathology. It permits the evaluation of the absence, presence and severity of these problems using a 7-point Likert scale (1 = absent to 7 = extreme). The instrument showed good internal consistency, with Cronbach’s alpha coefficients that range from 0.73 (positive symptoms) to 0.83 (negative symptoms), and from 0.96 (negative symptoms) to 0.99 (positive symptoms) for Southern Europe, including Romania [42].

#### 2.2.2. The White Bear Suppression Inventory (WBSI)

The WBSI [43] is a 15-item self-report scale that evaluates the general tendency to suppress unwanted negative thoughts, using a 5-point Likert scale (1 = strongly disagree to 5 = strongly agree). Some item examples are “I often do things to distract myself from the thoughts”, “I wish I could stop thinking of certain things” or “I have thoughts that I cannot stop”. The WBSI demonstrated very good internal consistency, with a Cronbach’s alpha of 0.88 [44]. WBSI was adapted for the Romanian population and acknowledged as a reliable measure of thought suppression, presenting a positive correlation with depressive symptoms [45].

#### 2.2.3. The Automatic Thoughts Questionnaire (ATQ)

The ATQ [46] is a 30-item self-report questionnaire that measures the occurrence and frequency of negative self-statements associated with depression and negative emotions. Each item states a single thought, such as “My future is bleak” or “I’m worthless”. Participants are asked to rate the frequency of the thought on a 5-point Likert scale (from 1 = not at all to 5 = all the time), and total scores are obtained by summing the scores of the participants’ items. Regarding psychometric properties, the instrument presented Cronbach’s alphas coefficients of 0.96 and 0.92 for the classical and shorter 8-item version, respectively [47]. The reliability of the ATQ was adequate in a Romanian population [48].

### 2.3. Data Analysis

First, means and standard deviations were computed for each variable included in the study. Further, to investigate group differences in the severity of psychosis, thought suppression, and automatic thoughts, while controlling for participants’ age, a multivariate analysis of covariance (MANCOVA) was conducted. Finally, to test the relationships between study variables, we performed a separate series of Pearson’s r correlations for each group (PPD vs. SMIPD). Statistical analyses were conducted using IBM SPSS Statistics, version 20 (IBM Corp., Armonk, NY, USA).

## 3. Results

### 3.1. Demographics

Demographic characteristics are summarized in Table 1 for both groups. Results revealed that there were no significant differences between participants in the PPD group and those in the SMIPD group with respect to gender (χ^2^ (1, N = 55) = 1.51, *p* = 0.169), education (χ^2^ (3, N = 55) = 5.62, *p* = 0.131), or days of hospitalization (t (53) = −1.60, *p* > 0.05), with the exception of age, which yielded a significant difference (t (53) = 2.56, *p* < 0.05).

Specifically, participants in the PPD group were older than those included in the SMIPD group (Mean difference = 4.06, 95% CI (0.88; 7.23]). Thus, age was used as a covariate in the MANCOVA analysis.

### 3.2. Preliminary Analyses

Means and standard deviations for study variables included in the study are presented in Table 2.

### 3.3. Differences between PPD vs. SMIPD

A MANCOVA was conducted to examine the group differences in psychosis severity, thought suppression, and automatic thoughts (with age included as a covariate in the analysis). A significant difference emerged between the two groups (Wilks’ Lambda = 0.634), F (3, 50) = 9.602, *p* < 0.001. Participants in the PPD group reported a significant increased level of suppression of thoughts compared to participants included in the SMIPD group (F (1, 52) = 14.277; *p* < 0.001, partial η2 = 0.215). No other significant differences emerged between the two groups (*p* > 0.05) (for more details, see Table 2).

### 3.4. Relations between Thought Suppression and the Other Variables in the Two Groups

Finally, a series of Pearson’s r correlations were conducted for each group to explore the relationship between thought suppression and the other variables included in the study. For the PPD group, results showed a significant positive correlation between the level of thought suppression and negative automatic thoughts (r = 0.60, *p* ≤ 0.001), (see Table 3). For the participants in the SMIPD group, results showed a significant positive correlation between the level of thought suppression and negative automatic thoughts (r = 0.71, *p* ≤ 0.001) (see Table 4).

## 4. Discussion

Previous research conducted in various countries has approached thought suppression from the perspective of coping/metacognitive mechanisms and showed that this strategy occurred in individuals with psychotic disorders, especially with schizophrenia [32,33,34,35,36,37,38]. However, to our knowledge, there are no studies to date which have explored thought suppression as a cognitive strategy used by patients with FEP in a Romanian population, in two nosologically different diagnoses, PPD and SMIPD. Additionally, the relation between thought suppression and negative automatic thoughts was never investigated in these two particular types of psychosis (PPD vs. SMIPD) during FEP, in a Romanian population.

In this regard, our results showed that there is a significant difference between patients with PPD and SMIPD during FEP with respect to the use of thought suppression as a cognitive strategy. More specifically, patients with PPD reported higher scores on thought suppression compared to patients with SMIPD, pointing out that patients with PPD rely more on this cognitive strategy in the face of FEP as compared to patients with SMIPD. A possible explanation for this result is that patients with PPD have less insight in relation to their psychotic experience, in contrast to SMIPD patients [2]; thus, they might have reacted more negatively towards their aversive internal symptoms. Indeed, research has shown that it is common for individuals with psychotic symptoms that show lower levels of insight to respond negatively towards their symptoms (e.g., a poor response at one’s own thoughts as a consequence of a poor understanding about their thoughts and condition), while those with higher levels of insight to better react towards and cope with their symptoms (e.g., a better response at one’s own thoughts as a consequence of a better understanding about their thoughts and condition) [49]. Consequently, this might also be the case for individuals with PPD in our study (with potential less insight according to previous studies) [2] who might be more prone to react negatively towards their thoughts (more thought suppression) in comparison with SMIPD individuals (less thought suppression). However, future studies are needed to further examine why patients with PPD rely more on thought suppression than patients with SMIPD.

Furthermore, in our study we found a significant positive correlation between thought suppression and individuals’ negative automatic thoughts within the two groups. That is, the more individuals (with PPD and SMIPD) used thought suppression, the more they experienced an increase in their automatic negative thoughts. This is especially important in the current study, as both individuals with PPD and SMIPD showed this tendency, which might further complicate/exacerbate their condition. While suppression of thoughts might be effective in dealing with FEP in the short term, this might come with an associated negative cost/complication (i.e., the experience of negative automatic thoughts). Thus, we suggest that instructing individuals on more effective coping strategies, such as reappraisal or finding the meaning related to their symptoms, can help individuals with PPD and SMIPD to better cope with the FEP and with associated emotional symptoms. To our knowledge this is the first study to capture these relations in a Romanian sample of individuals with psychotic symptoms. Thus, treatments could address thought suppression in patients with psychotic symptoms in order to prevent further complications (i.e., the experience of negative automatic thoughts). For this purpose, alternatives should be sought to ensure access to psychotherapy services for this population. Internet-based cognitive behavioral therapy (iCBT) is a tailored intervention designed to deliver psychotherapy via online services for individuals with various clinical conditions. Preliminary evidence suggests that iCBT could be also beneficial for patients with psychosis and schizophrenia [50,51]. Therefore, different cognitive techniques for psychosis, such as normalizing thoughts and psychotic experiences of patients, working to decrease the negative consequences of voices and hallucinations, as well as using behavioral experiments, may be easily integrated in iCBT interventions, aiming to reduce thought suppression in individuals with psychotic symptoms. In this regard, a viable solution is using an online educational platform, for example Moodle e-learning platform [52], which has already proved to be useful in delivering online iCBT services for patients with various forms of mental disorders [53]. As a result, iCBT may be used for targeting thought suppression in psychosis on the Moodle e-learning platform, in three ways: (1) the use of cognitive techniques described above; (2) psychoeducation regarding thought suppression, and (3) real-time assessment of the frequency and intensity of thought suppression, using specific worksheets. Consequently, iCBT could be a suitable, cost-effective, non-pharmacological treatment for multiple psychiatric disorders, including psychotic disorders [53]. Finally, current results point towards the multiple interrelated mechanisms that might be involved in the development, maintenance, and exacerbation of this condition, as well as towards the need for multimodal interventions when addressing it in the therapeutic process.

### Clinical Implications

Cognitive behavioral therapy (CBT) is considered a psychological intervention which can be effectively used in the treatment of a psychotic disorder [54,55]. Therefore, knowing that thought suppression appears during FEP, especially in patients with PPD, early cognitive behavioral therapy for psychosis (CBTp) based on cognitive restructuring (e.g., targeting the decreasing of the level of thought suppression), could be used to improve patients’ conditions. At the same time, cognitive interventions may be helpful to decrease the negative emotions caused by the negative automatic thoughts, both in patients with PPD and SMIPD during FEP. Psychoeducation serves as an opportunity to normalize and decatastrophize the experience of psychotic symptoms, which is why physicians are trained to use this technique at an early stage in their clinical practice [56].

## 5. Limitations

Our study has some notable limitations. First, it was difficult to establish a clear diagnosis for the PPD group. PANNS was conducted immediately after the psychiatric consultation, and the application of psychological questionnaires was at about 7–12 days after PANNS, which means that the time was too short to establish a definitive and specific diagnosis (i.e., brief-episode psychosis, schizophrenia, or schizoaffective disorder). Second, even though depression and anxiety are often comorbid with psychosis, and thought suppression is common among individuals with depression and/or anxiety, we have not assessed/investigated this comorbidity in our study. Third, both groups included a small number of participants (≤30). Fourth, another limitation is related to the lack of a control group which prevented our interpretative ability. Specifically, future studies should include a healthy control group to better determine specific difficulties for individuals with FEP in comparison with healthy individuals. Fifth, another important limitation is related to the measure we used in the current study to assess thought suppression. While this scale is a well-established measure in the empirical literature, it does not address specifically the suppression of thoughts that are related to psychotic symptoms in particular. Hence, it would have been clearer to use a scale that includes items assessing the suppression of thoughts related to psychotic symptoms (delusional thoughts). Finally, patients completed the questionnaires in the early stages of the disorder and under the influence of antipsychotic medication. Future research should overcome these limitations, providing a more accurate and comprehensive perspective on this phenomenon.

## 6. Conclusions

In conclusion, FEP patients with PPD use thought suppression more, as opposed to SMIPD, where the onset of psychosis is sudden, lacking prodromal manifestation, generally immediately following substance/medication consumption. As a typical thought-control strategy in psychosis, suppression can lead to a paradoxical increase in the frequency of intrusive thoughts and associated negative automatic thoughts. The current study supports the empirical evidence indicating that thought suppression may be viewed as an unhealthy reaction to psychotic experiences due to its association with negative automatic thoughts both in patients with PPD and patients with SMIPD.

## Figures and Tables

**Table 1:** Demographic Description of Characteristics of participants from PPD and SMIPD.

Sample Characteristics	Individuals with Primary Psychotic Disorder (PPD) (*n* = 30)	Individuals with Substance/Medication Induced Psychotic Disorder (SMIPD) (*n* = 25)	Statistical Significance (*χ^2^*/*t*)	*p*
Gender *n* (%) female, *χ^2^*	17 (56.7)	10 (40)	1.51	0.169
Age range, *M* (*SD*), *t*	18–44 27.30 (6.92)	18–31 23.24 (4.16)	2.56	0.013
Education *n* (%), *χ^2^*			5.62	0.131
Primary School	3 (10)	0 (0)		
Middle school	6 (20)	11 (44)		
High School	12 (40)	7 (28)		
Undergraduate	9 (30)	7 (28)		
Days of hospitalization, *M* (*SD*), *t*	17.90 (8.83)	10 (40)	−1.60	0.114

Note. *M* = mean; *SD* = Standard Deviation.

**Table 2:** Descriptive Statistics for Psychosis Severity, Suppression, and Automatic Thoughts.

Measure	Individuals with Primary Psychotic Disorder (PPD) (*n* = 30) *M* (*SD*)	Individuals with Substance/Medication Induced Psychotic Disorder (SMIPD) (*n* = 25) *M* (*SD*)	Statistical Significance (*F*)	Partial *η2*
PANSS	90.50 (3.79)	89.20 (5.14)	*F* (1, 52) = 0.995; *p* = 0.323	0.019
ATQ	28.23 (16.04)	27.28 (13.96)	*F* (1, 52) = 0.384; *p* = 0.538	0.007
WBSI	49.83 (15.71)	31.80 (13.21)	*F* (1, 52) = 14.277; *p* < 0.001	0.215

Note. PANSS = The Positive and Negative Syndrome Scale; ATQ = The Automatic Thoughts Questionnaire; WBSI = The White Bear Suppression Inventory; age was included as a covariate in the analysis; M = Mean; SD = Standard Deviation.

**Table 3:** Zero-order correlations between the variables in the study for Individuals with Primary Psychotic Disorder (PPD).

Variable	1	2	3
1. PANSS	-	−0.212	−0.166
2. ATQ		-	0.609 **
3. WBSI			-

Note. PANSS = The Positive and Negative Syndrome Scale; ATQ = The Automatic Thoughts Questionnaire; WBSI = The White Bear Suppression Inventory; ** *p* ≤ 0.001.

**Table 4:** Zero-order correlations between the variables in the study for Individuals with Substance/Medication Induced Psychotic Disorder (SMIPD).

Variable	1	2	3
1. PANSS	-	0.233	0.142
2. ATQ		-	0.717 **
3. WBSI			-

Note. PANSS = The Positive and Negative Syndrome Scale; ATQ = The Automatic Thoughts Questionnaire; WBSI = The White Bear Suppression Inventory; ** *p* ≤ 0.001.

## Data Availability

The data presented in this study are available on request from the corresponding author (razvanpredatu@psychology.ro).
